# Cholinergic and Noradrenergic Modulation of Corticothalamic Synaptic Input From Layer 6 to the Posteromedial Thalamic Nucleus in the Rat

**DOI:** 10.3389/fncir.2021.624381

**Published:** 2021-04-26

**Authors:** Syune Nersisyan, Marek Bekisz, Ewa Kublik, Björn Granseth, Andrzej Wróbel

**Affiliations:** ^1^Nencki Institute of Experimental Biology, Polish Academy of Sciences, Warsaw, Poland; ^2^Department of Clinical and Experimental Medicine, Linköping University, Linköping, Sweden; ^3^Faculty of Philosophy, University of Warsaw, Warsaw, Poland

**Keywords:** gain control, *in vitro*, intracellular recordings, frequency-dependent facilitation, cholinergic and noradrenergic modulation

## Abstract

Cholinergic and noradrenergic neuromodulation of the synaptic transmission from cortical layer 6 of the primary somatosensory cortex to neurons in the posteromedial thalamic nucleus (PoM) was studied using an *in vitro* slice preparation from young rats. Cholinergic agonist carbachol substantially decreased the amplitudes of consecutive excitatory postsynaptic potentials (EPSPs) evoked by a 20 Hz five pulse train. The decreased amplitude effect was counteracted by a parallel increase of synaptic frequency-dependent facilitation. We found this modulation to be mediated by muscarinic acetylcholine receptors. In the presence of carbachol the amplitudes of the postsynaptic potentials showed a higher trial-to-trial coefficient of variation (CV), which suggested a presynaptic site of action for the modulation. To substantiate this finding, we measured the failure rate of the excitatory postsynaptic currents in PoM cells evoked by “pseudominimal” stimulation of corticothalamic input. A higher failure-rate in the presence of carbachol indicated decreased probability of transmitter release at the synapse. Activation of the noradrenergic modulatory system that was mimicked by application of norepinephrine did not affect the amplitude of the first EPSP evoked in the five-pulse train, but later EPSPs were diminished. This indicated a decrease of the synaptic frequency-dependent facilitation. Treatment with noradrenergic α-2 agonist clonidine, α-1 agonist phenylephrine, or β-receptor agonist isoproterenol showed that the modulation may partly rely on α-2 adrenergic receptors. CV analysis did not suggest a presynaptic action of norepinephrine. We conclude that cholinergic and noradrenergic modulation act as different variable dynamic controls for the corticothalamic mechanism of the frequency-dependent facilitation in PoM.

## Introduction

In addition to afferent sensory thalamocortical fibers, the thalamic cells of mammals are reached by feedback corticothalamic axons that outnumber the peripheral projection (Rouiller and Welker, 2000). The major source of this descending feedback input to the thalamus originates in the pyramidal neurons of the cortical layer 6 (Erisir et al., 1997; Alitto and Usrey, 2003; Sherman and Guillery, 2006; Van Horn and Sherman, 2007). The layer 6 input evokes direct depolarization of the thalamic relay cells (Lindström and Wróbel, 1990; Reichova and Sherman, 2004) or indirect hyperpolarization via recurrent interneurons in the thalamic reticular nucleus (Lam and Sherman, 2010). One hypothesis regarding the layer 6 input to the thalamus posits its functional role as a variable gain regulator for sensory relay at the thalamus. This mechanism would control the flow of ascending sensory information from the periphery to the cortex depending on the behavioral state of the animal (Ahlsen et al., 1985; Lindström and Wróbel, 1990; Granseth et al., 2002; Granseth, 2004; Lam and Sherman, 2010).

In the rat somatosensory system both the first-order ventrobasal nucleus (VB) and the higher-order posteromedial nucleus (PoM) receive cortical input from layer 6. PoM, however, receives an additional driver input from cortical layer 5. Accordingly, PoM is thought to be involved in cortico-cortical transmission via a cortico-thalamo-cortical route (Theyel et al., 2010).

Sensory thalamus and sensory cortex are extensively innervated by rich cholinergic and noradrenergic neuromodulatory inputs from the brainstem and basal forebrain. Most of the studies on these modulatory systems demonstrate their role in setting different vigilance levels from awakening to arousal (Steriade et al., 1993). While their role in sleep-wake cycles is well recognized, much less is known about the mechanisms underlying the neuromodulatory action at sensory relays. Specifically, we are not aware of any research investigating the modulation of synaptic integration at higher-order thalamic nuclei, even though they receive denser modulatory projections than the first-order nuclei (Van Horn and Sherman, 2007). Sensory thalamus receives powerful modulatory projections from the brainstem and from layer 6 of the primary sensory cortex (Erisir et al., 1997). Importantly, the cortical layer 6 synaptic input to the thalamic neurons is also efficiently regulated by projections from the brainstem (Steriade, 2000; Castro-Alamancos and Calcagnotto, 2001). The interplay between the cortical and brainstem modulatory inputs may constitute a complex functional control system of the thalamic cells. Therefore, our study aimed to investigate the influence of cholinergic and noradrenergic modulatory systems on the synaptic transmission from cortical layer 6 to the higher-order somatosensory posteromedial thalamic nucleus, with special focus on facilitation at the corticothalamic synapse.

## Materials and Methods

### Preparation of Slices

All experiments were performed with the approval of the first Local Ethic Commission in Warsaw and Committee for Ethics in Animal Research of Linköping in accordance with Polish, Swedish and EU legislations.

**FIGURE 1:**
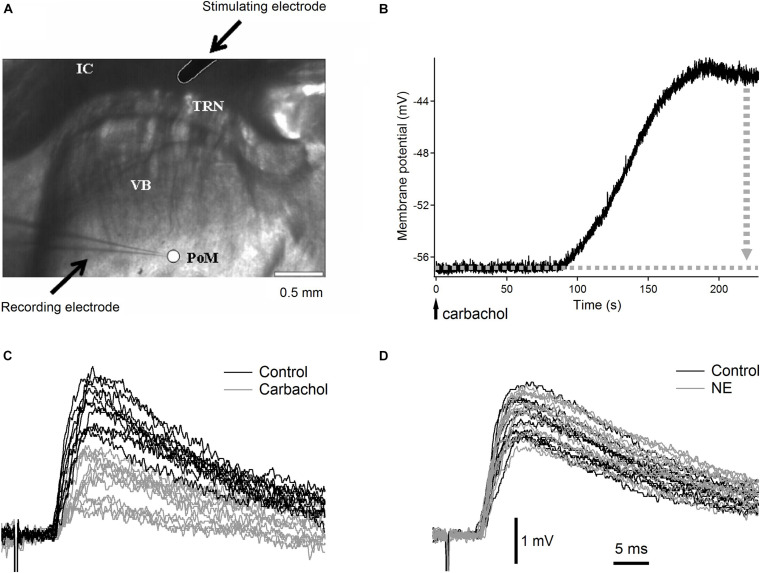
Raw experimental data. **(A)** Low magnification (1.4×) image of the somatosensory thalamic area with recording electrode in posteromedial nucleus and stimulation electrode in the internal capsule. VB, ventrobasal nucleus with characteristic stripes of dense fibers; PoM, posteromedial nucleus recognized as uniform, brighter area; IC, internal capsule; TRN, thalamic reticular nucleus; arrows point to recording and stimulating electrodes. Scale bar is 0.5 mm. **(B)** Typical depolarization of a PoM cell after application of cholinergic agonist carbachol. The vertical dotted gray arrow indicates the manual compensation of membrane potential shift by adding negative DC current. **(C)** Examples of raw EPSPs evoked in a PoM cell by the 1st impulse of the five-pulse train (0.033 Hz train repetition rate) stimulating cortico-thalamic axons in the internal capsule. Black traces show EPSPs in the control condition (with the ACSF containing both GABA_*A*_ and GABA_*B*_ inhibitors – 10 μM bicuculline and 2 μM CGP55845 respectively). Gray traces show EPSPs after adding carbachol (7 μM) to the ACSF. **(D)** Raw EPSPs evoked by the 1st stimulation pulse in the control condition (black traces) and after adding norepinephrine (100 μM) to the ACSF (gray traces). In this case, as with the study of each noradrenergic substance in this work, both control and test ACSFs contained in addition to GABA_*A*_ and GABA_*B*_ inhibitors also an antioxidant – 40 μM sodium ascorbate. In **(C,D)** all EPSPs were recorded at similar membrane potential between –56 and –57 mV but their individual baselines varied within about 1 mV range. To better show inter-trial fluctuations of EPSPs amplitude, all EPSP traces were adjusted in **(C,D)** to the same baseline. Vertical and horizontal scales for **(C,D)** are indicated by black bars at the bottom of **(D)**.

Three- to four-week old Wistar rats were decapitated under deep isoflurane anesthesia. Brains were quickly removed and immersed in cold (between −1°C and +0.5°C) artificial cerebrospinal fluid (ACSF) with NaCl substituted with sucrose, having the following composition (in mM): KCl 3, NaH_2_PO_4_ 1.25, NaHCO_3_ 24, MgSO_4_ 4, CaCl_2_ 0.5, D-glucose 10, sucrose 219 (300–308 mOsm). Thalamocortical slices (350 μm) (Agmon and Connors, 1991; Land and Kandler, 2002), ideally suitable for selective studies of synapses formed on PoM cells by axons from cortical layer 6 (Landisman and Connors, 2007) were prepared using a Leica VT1000S vibrating blade microtome. Slices were incubated at 31°C for 30 min and then at room temperature for at least 1 h. ACSF in the incubation chamber contained (in mM): NaCl 126, KCl 3, NaH_2_PO_4_ 1.25, NaHCO_3_ 24, MgSO_4_ 3, CaCl_2_ 1, D-glucose 10. Individual slices were transferred to the recording chamber, with circulating (2–2.5 ml/min), warm (31–32°C) ACSF of a similar composition to the incubation solution except for MgSO_4_ and CaCl_2_ which concentrations were changed to 2 mM. All solutions were saturated with 95% O_2_–5% CO_2_. The recording chamber was mounted under the nosepiece of an Olympus BX61WI microscope equipped with a C7500 near infrared CCD video camera (Hamamatsu, Hamamatsu City, Japan). In most of the slices, the cortex was cut-off to prevent activation of the thalamo-cortico-thalamic loop. In the thalamocortical slices, the PoM nucleus was readily distinguished from the VB, TRN, and the internal capsule when using a low-magnification (4x) objective with an additional 0.35x magnification changer (1.4 × final magnification; Figure 1A).

### Pharmacology

Activation of cholinergic or noradrenergic modulatory system was mimicked by bath application of a non-specific cholinergic agonist carbamoylcholine chloride (carbachol) (6–8 μM) or norepinephrine hydrochloride (100 μM), accordingly. All drugs were added to the ACSF that perfused the slices and 3–5 min was allowed for complete solution-exchange in the recording chamber. This time period was determined from preliminary experiments with drugs that depolarized the neurons. Incubation with the drug lasted usually 5–25 min.

Bicuculline methiodide (10 μM) was used to block GABA_*A*_ receptors. For complete elimination of the recurrent inhibitory influence from the thalamic reticular nucleus (TRN) the GABA_*B*_ receptor antagonist CGP 55845 hydrochloride (2 μM) was also used in most of the experiments. The GABA receptor inhibitors were present during both the control period and periods of the application of cholinergic or adrenergic agents. To investigate the role of different subtypes of cholinergic and adrenergic receptors in the observed effects, the following specific receptor agonists and antagonists were used: nicotinic agonist DMPP (dimethylphenylpiperazinium, 10 μM); muscarinic receptor antagonist scopolamine (1 μM); adrenergic α-2 receptors agonist clonidine hydrochloride (40 μM), α-1 adrenergic receptors agonist phenylephrine hydrochloride (100 μM) and β adrenergic receptors agonist isoproterenol hydrochloride (100 μM). All chemicals were obtained from Sigma (St Louis, MO, United States), except for CGP 55845 hydrochloride which was purchased from Tocris (Bristol, United Kingdom). To prevent oxidation of adrenergic agonists, in the experiments where adrenergic agents were used, sodium ascorbate (40 μM) was present in the ACSF during both the control period and incubation with the drugs.

### Recording and Stimulation

Whole-cell patch-clamp recordings were performed from PoM neurons using electrodes (3–6 MΩ) pulled from standard-wall (1.2 mm outer diameter) borosilicate glass capillaries. In most of the experiments, electrodes were filled with (in mM): potassium gluconate 120, HEPES 10, EGTA 0.1, KCl 4, NaCl 2, Mg-ATP 4, Na_2_-GTP 0.5, phosphocreatine (Tris salt) 10; pH was adjusted to 7.25 with KOH and osmolarity to 285–290 mOsm with sucrose.

To improve the space constancy of the maintained membrane potential in the voltage clamp method during the experiments with the pseudominimal stimulation (see below), a Cs-based electrode solution was used with the following composition (in mM): Cs-gluconate 100, NaCl 10, HEPES 10, TEA-Cl 20, QX-314 5, EGTA 0.1 and Mg-ATP 1; pH = 7.3, osmolarity adjusted to 300 mOsm.

In most experiments, the membrane potential was recorded in fast current-clamp mode with Axopatch 200B amplifier and pCLAMP software (Molecular Devices, United States). In “pseudominimal stimulation” experiments the thalamus and PoM cells were visualized using Axioskop FS microscope (Carl Zeiss, Jena, Germany) with Hamamatsu C7500 camera and membrane current was recorded in voltage-clamp mode using Heka EPC9 (HEKA Elektronik, Lambrecht, Germany) amplifier and Pulse software. In current clamp, the recorded membrane potential values were not corrected for the junction potential. In voltage clamp, the holding membrane potential was corrected for the measured 8 mV junction potential. To evoke excitatory postsynaptic potentials (EPSPs) or currents (EPSCs), repetitive trains of five electrical pulses (200 μs duration) at 20 Hz frequency were applied through a concentric stimulating electrode placed at the corticothalamic fiber tract in the internal capsule (Figure 1A). Individual trains were repeated at 30 s interval. Stimulation current ranged from 90 to 500 μA. The electrical train stimuli were repeated 8–30 times in control condition and 16–50 times after corresponding cholinergic or noradrenergic drug application (see below).

Typically, after application of most cholinergic or noradrenergic agonists to the ACSF, the neurons started to depolarize after about 60–90 s and reached a steady state after 100 s. For instance, application of carbachol and norepinephrine depolarized PoM neurons by 9.1 ± 1.3 mV and 11.8 ± 0.7 mV (mean ± SEM; *n* = 16 and *n* = 15, correspondingly) (Figure 1B). If necessary, the depolarization induced by cholinergic or noradrenergic agents was compensated by manual injection of a negative DC current through the recording electrode which brought the membrane potential back to a more negative value. This compensation was done in order to have the same driving force for the ions responsible for the generation of the EPSPs in the control condition and after application of the appropriate drug. To avoid the appearance of low-threshold calcium spikes during the stimulation train, EPSPs were recorded in all situations at adjusted manually membrane potential of –56 mV (with an accuracy of ±1 mV). The membrane resistance was measured always at resting potential from the initial recording period prior to administration of the agonists.

During the voltage-clamp pseudominimal stimulation experiments, the stimulation intensities were adjusted to activate a sufficiently low number of corticothalamic axons so that the initial postsynaptic responses had a failure rate of ∼50%. These EPSC recordings were made at a holding potential of –58 mV. In this kind of experiment, the stimulation train was repeated 35–68 times in control and 47–158 times after drug application.

### Analysis and Statistics

Excitatory postsynaptic potentials amplitudes were measured from the baseline to the peak amplitude (in pCLAMP). In case of temporal overlap during train stimulation, the decay of the preceding EPSP was exponentially extrapolated and used as a baseline for measuring the amplitude of the consecutive EPSPs. In order to examine facilitation of consecutive responses in trains EPSP amplitudes were normalized to the first EPSP amplitude (EPSP_*n*_/EPSP_1_; “normalized amplitudes”). Ratios between amplitudes of successive EPSPs (EPSP_*n*_/EPSP_*n–*__1_) were also calculated to illustrate the temporal (instantaneous) changes of facilitation during stimulation trains.

The coefficients of variations (CVs) of the noise-free inter-trial amplitude fluctuations of the consecutive postsynaptic potentials were estimated from the data as the square root of the noise-free variance of the EPSP amplitude distribution, divided by the mean EPSP amplitude (Clements, 1990). An exemplary inter-trial variation of the amplitudes of the 1st EPSP in various experimental conditions can be traced in Figures 1C,D. In the noise-free variance calculation, the variance of the noise was subtracted from the variance of the individual EPSP amplitudes. Consequently, CV values were calculated according to the following equation:

(Eq. 1) $$\mathrm{CV}={\left(\mathrm{Var}\,\left(\mathrm{EPSP}\right)-\mathrm{Var}\,\left(\mathrm{noise}\right)\right)}^{1/2}/\mathrm{Mean}\,\left(\mathrm{EPSP}\right)$$

The CV values calculated in the control condition were compared to the CVs during drug exposure. Large differences between CVs in these two conditions strongly implicated a presynaptic site of modulation (Clements, 1990; Nagumo et al., 2011).

Analysis of EPSCs recorded in voltage-clamp was performed with IgorPro (Wavemetrics Inc., United States). The EPSC amplitudes were measured as the difference between the mean membrane current over 1.5 ms at the peak of the EPSC and the preceding 1.5 ms baseline. Noise distribution was obtained by similar measurements during the baseline period. EPSC amplitudes smaller than 2SD of the noise distribution were considered to be EPSC failures (Granseth and Lindström, 2003). The SD at the noisiest condition for each cell was used for this classification whether it was recorded from control condition or with carbachol. The EPSC failure (or response success) rates were calculated as the number of failures (or responses) divided by the total number of stimulation trains (N_*failures*_/N_*trains*_ or N_*responses*_/N_*trains*_) for each pulse in each cell individually. EPSC amplitude histograms were constructed using a bin size of 0.5 pA and accumulated across cells. To determine the quantal size (Q) of the corticothalamic EPSCs, the averaged amplitude probability histograms obtained for the first impulse were fitted with a double Gaussian function with two peaks separated from zero with Q and 2Q and the same standard deviation (del Castillo and Katz, 1954).

Throughout the text, the averaged data were presented as means ± SEM. Student *t*-test for paired comparisons was used throughout the text, unless otherwise indicated and *P* ≤ 0.05 was considered to be significant. In case of multiple comparisons, significance of P values was additionally checked with Benjamini–Hochberg (B-H) false discovery rate (FDR) procedure at level 0.05 (Benjamini and Hochberg, 1995). According to a suggestion given by McDonald (2014) we did not present B-H FDR corrected *P*-values. Instead, we show the original *P*-values and describe which remain significant after using B-H FDR procedure.

### Histological Staining

Some slices were subjected to cytochrome oxidase histochemistry to visualize the somatosensory thalamic nuclei, i.e., to highlight the border between VB and PoM. For this purpose, slices were fixed in 4% formalin, washed with phosphate buffer (0.05M, pH = 7.4) and incubated in DAB solution (100 ml of which contained: sucrose 1 g, DAB 25 mg, cytochrome C 15 mg, catalase 10 mg, imidazole 250 μl, nickel ammonium sulfate 50 mg) on a shaker at 30–40°C for about 2–3 h until specific staining appeared. Finally, slices were rinsed in a phosphate buffer three times, 5 min each. After the staining, images of the stained and non-stained slices were compared to confirm the localization of the recorded cells.

## Results

### Basic Electrophysiological Properties of PoM Cells

In the vast majority (≈90%, *n* = 74) of the investigated cells the membrane potential was recorded in current-clamp mode. The average resting membrane potential was –61.92 ± 0.31 mV and the membrane resistance was 84.90 ± 3.36 MΩ, which slightly differ from those described earlier (Landisman and Connors, 2007). In particular, a little less negative membrane potential and a little larger membrane resistance was reported by these authors. This discrepancy may result from the age difference of the experimental animals (3–4-week-old in our experiments versus 2–3-week-old used by Landisman and Connors).

In response to the injection of 500 ms rectangular depolarizing or hyperpolarizing current pulses, the firing pattern of PoM neurons exhibited tonic and burst modes typical for thalamic cells (Jahnsen and Llinas, 1984). In the tonic mode, the response during a +300 pA depolarizing pulse was characterized by mean firing rate of 39.5 ± 6.7 Hz and in burst mode the response to a –200 pA hyperpolarizing current was characterized by mean burst frequency of 265 ± 17.3 Hz. This burst firing frequency is similar to the one reported for PoM neurons by Landisman and Connors (2007), however much lower than the value obtained for VPM cells by the same authors. This fact additionally supports the notion that the cells recorded in our experiments were located in PoM.

Facilitation of the EPSP amplitudes is a typical feature of corticothalamic synapses formed by axons descending from layer 6 pyramids to thalamic relay cells (Lindström and Wróbel, 1990; Granseth et al., 2002; Granseth, 2004). That is, with high frequency (i.e., 20 Hz) stimulation train the first impulse evokes an EPSP of a small amplitude while the amplitudes of the EPSPs evoked by the following pulses in the train are progressively enhanced. The opposite effect characterizes layer 5 input when the first impulse evokes a large EPSP while following responses in a high-frequency train are progressively decreased (Reichova and Sherman, 2004; Groh et al., 2008). We observed synaptic facilitation in response to a 20 Hz stimulation in all recorded PoM cells. This proved that “classical” thalamocortical slices (Agmon and Connors, 1991; Land and Kandler, 2002) used by us were well suited for selective studies of the synapses formed on PoM cells by the axons from cortical layer 6 (Landisman and Connors, 2007), as in such preparation the corticothalamic fibers from layer 5 appeared to be mostly cut.

### Cholinergic and Noradrenergic Systems Differentially Modulate Corticothalamic Synaptic Transmission in PoM

**FIGURE 2:**
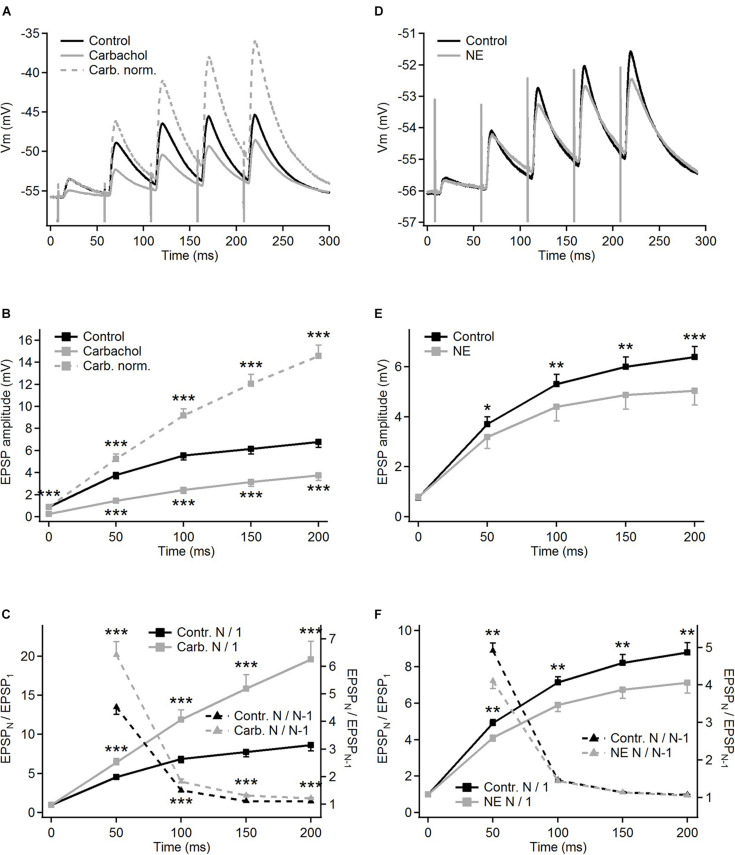
Modulation of frequency-dependent facilitation of the cortico-thalamic synapse in PoM by cholinergic and noradrenergic agents. **(A)** Examples of facilitating synaptic responses of a single PoM cell to the electrical stimulation of the cortico-thalamic axons in control condition with ACSF containing GABA inhibitors (black trace, average of 11 trials), and after adding 6–8 μM carbachol (solid gray trace, average of 33 trials). Dashed gray trace shows the data with carbachol after normalization to the first control EPSP. **(B)** Average amplitudes of the consecutive EPSPs in the train, measured for a group of 16 cells studied with carbachol. Mean amplitudes obtained for “carbachol” (solid gray line) conditions were significantly lower than in “control” (black line) for each EPSP in the train. **(C)** Left *Y* axis: normalized amplitudes (EPSP_*N*_/EPSP_1_, the same *n* = 16 cells) in control conditions (solid black trace) and in the presence of carbachol [solid gray line; note that the same data are included in **(B)** as gray dashed line]. Right *Y* axis: momentary facilitation, i.e., ratios of the consecutive EPSP amplitudes (EPSP_*N*_/EPSP_*N–*__1_) in control conditions and after application of carbachol (dashed lines). Both measures were significantly different from control values for all EPSP in the train. **(D)** Examples of the averages of single cell postsynaptic responses to stimulation of the corticothalamic axons in the control condition with GABA inhibitors and ascorbic acid (black trace, 22 repetitions) and after adding 100 μM norepinephrine (NE, gray trace, 32 repetitions). **(E)** Average amplitudes of the consecutive EPSPs measured for a whole group of cells (*n* = 15) studied with norepinephrine. Black trace shows EPSP amplitudes in control condition, gray trace – after adding norepinephrine. **(F)** Left *Y* axis: average normalized EPSP amplitudes (EPSP_*N*_/EPSP_1_) in the control condition (black trace) and after application of norepinephrine (gray trace). Right *Y* axis: average ratios of the neighboring EPSP amplitudes (EPSP_*N*_/EPSP_*N–*__1_) in the control condition and after application of norepinephrine (black and gray dashed lines, respectively). Data are expressed as mean ± SEM, *P ≤ 0.05, **P ≤ 0.01, ***P ≤ 0.001.

Compared to the control condition, carbachol substantially decreased the amplitudes of all postsynaptic responses evoked by five impulses of a 20 Hz electrical stimulation of the corticothalamic axons. The amplitude reduction (about threefold) was most pronounced for the first EPSP in the train (Figure 2A, gray trace; and raw, non-averaged potential waveforms in Figure 1C). The following postsynaptic potentials were affected progressively less than the first one. Consequently, the amplitude of the last EPSP in the presence of carbachol was less than two times smaller than the one recorded in the control condition. Apparently, in parallel to the reduction of the EPSP amplitudes, carbachol increased the facilitation of the EPSPs during the 5-pulse train stimulation (see normalized amplitudes in Figure 2A).

For the group of the studied cells (*n* = 16), the mean amplitude of the first EPSP was 3.4 times smaller after application of carbachol (Figure 2B – gray vs. black trace). Much weaker responses were also evoked by the subsequent pulses in the train. The relative reduction of the 2nd, 3rd, 4th, and 5th EPSP became progressively smaller (for appropriate numerical values see Table 1A), supporting the idea that despite the reduction of the amplitudes of all EPSPs in the train the application of carbachol increased the facilitation of consecutive responses.

**TABLE 1:** Cholinergic effects.

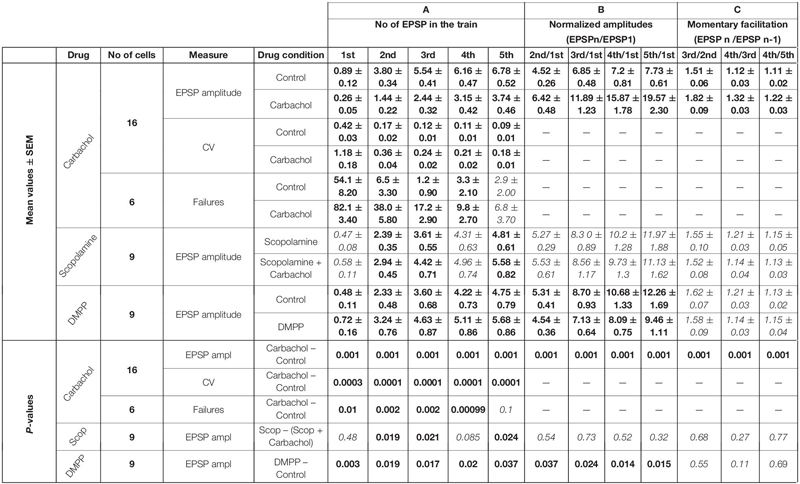

*Measured values and their corresponding *p*-values that were significant are written in bold, nonsignificant in faint italic. Abbreviations: ampl – amplitude; Scop – Scopolamine.*

Facilitation of consecutive EPSPs in the control condition and in the presence of carbachol was in addition analyzed by calculating normalized amplitudes (each EPSP amplitude in the train divided by the first one – EPSP_*N*_/EPSP_1_) (Figure 2C). In the presence of carbachol normalized amplitude of the 2nd EPSP was 1.42 times larger than the one obtained during the control condition. Normalized amplitudes of the 3rd, 4th, and 5th EPSPs obtained for carbachol were accordingly 1.73; 2.05 and 2.27 times larger than in control (Figure 2C, see also Table 1B). These results indicate that carbachol induced a consistent and instantaneous increase of facilitation along the train of consecutive EPSPs. In the presence of carbachol the 5th EPSP had about 20 times larger amplitude than the 1st EPSP. This ratio (2.27 times larger than for the control situation) demonstrates the potency by which carbachol enhances the global facilitation of EPSP amplitudes during the 5 impulses/20 Hz stimulation train.

The examination of the momentary changes in facilitation along the train (calculated by EPSP_*N*_/EPSP_*N–*__1_ ratio and plotted in Figure 2C as dashed lines against the right vertical axis) revealed that although the largest increase of the EPSP_*N*_/EPSP_*N–*__1_ ratio occurred for the first two EPSPs (2nd/1st = 1.42) the carbachol-induced enhancement of momentary facilitation affected also the subsequent responses in the train (the 3rd/2nd ratio was 1.20 times larger than in control condition; 4th/3rd by 1.18; 5th/4th by 1.10; *P* < 0.001 for each pair of comparisons, all significant using FDR procedure at level 0.05; see also Table 1C).

In contrast to carbachol, norepinephrine did not change the amplitude of the first EPSP in the train (Figures 1D, 2D), however, it did reduce the amplitudes of later EPSPs (Figure 2D). Similar to a single cell observation, norepinephrine did not change the mean amplitude of the 1st EPSP for a group of PoM cells studied with this drug (*n* = 15), but reduced amplitudes of the 2nd, 3rd, 4th, and 5th EPSPs (Figure 2E and Table 2A).

**TABLE 2:** Noradrenergic effects.

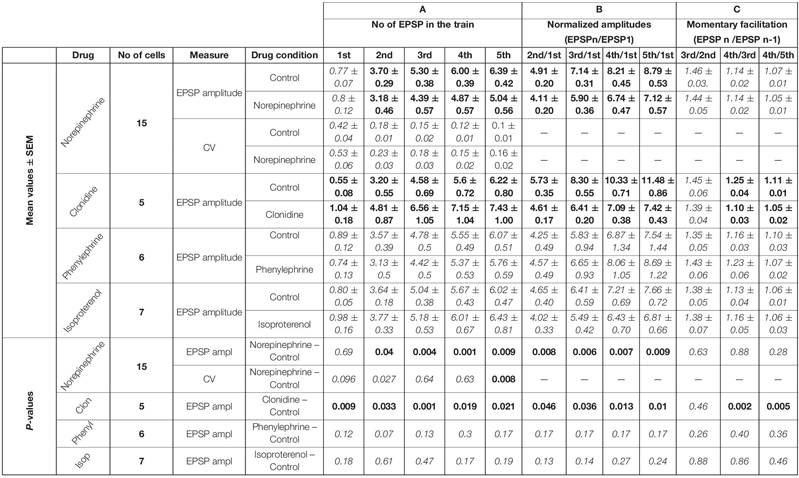

*Measured values and their corresponding *p*-values that were significant are written in bold, nonsignificant in faint italic. Abbreviations: ampl – amplitude; Clon – Clonidine, Phenyl – Phenylephrine; Isop – Isoproterenol.*

In general, the amplitude reduction caused by norepinephrine suggests a moderate decrease in facilitation during the train. Indeed, the average values of the normalized amplitudes (EPSP_*N*_/EPSP_1_) (Figure 2F and Table 2B), were consistently larger in the control condition than in the presence of norepinephrine indicating a decrease of the facilitation after application of the drug. Normalized amplitudes obtained for the 2nd, 3rd, 4th, and 5th EPSP were by 16.3, 17.4, 17.9, and 19% smaller in the presence of norepinephrine. Trend for a higher reduction for late EPSPs was, however, weak and the observed decrease of facilitation was solely due to the difference in ratios between the amplitudes of the 2nd and the 1st EPSPs. The momentary facilitation (EPSP_*N*_/EPSP_*N–*__1_) differed only for the first pair of the postsynaptic responses (EPSP_2_/EPSP_1_, *P* = 0.008) (Figure 2F). The following ratios (3rd/2nd, 4th/3rd, 5th/4th EPSP) measured in control condition and after application of norepinephrine were similar (see Table 2C).

To sum up, activation of cholinergic receptors by carbachol significantly reduced the amplitudes of all EPSPs evoked in PoM cells by train stimulation of descending fibers from the cortical layer 6, simultaneously enhancing the frequency-dependent facilitation at this synapse. Instead, activation of noradrenergic receptors via application of norepinephrine decreased the amplitudes of all but the first EPSP evoked by train stimuli, indicating a reduction of the facilitation.

### Muscarinic Receptors Are Responsible for Cholinergic Modulation of Corticothalamic EPSPs

**FIGURE 3:**
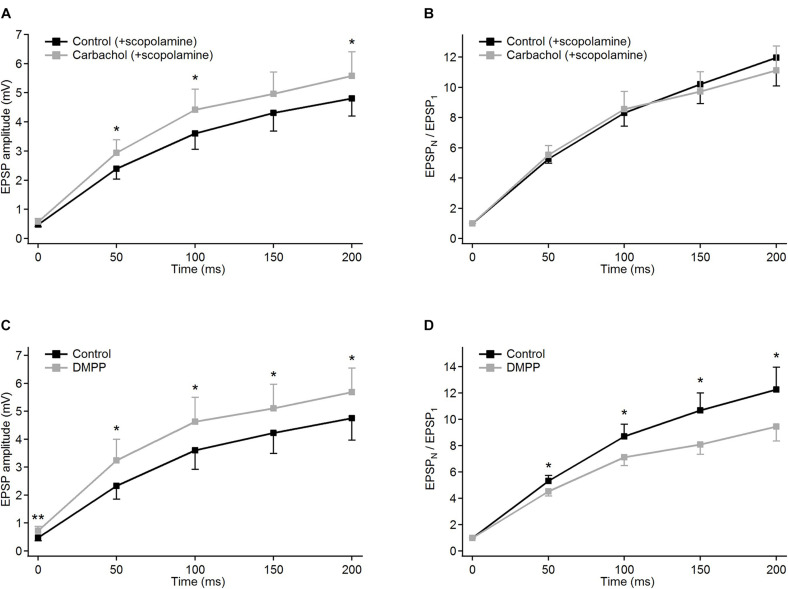
Modulatory effects of different cholinergic agents on EPSP trains in PoM cells. **(A,B)** Blockade of muscarinic receptors by scopolamine: **(A)** EPSP amplitudes and **(B)** corresponding normalized EPSP amplitudes averaged for nine cells in the control condition (ACSF containing GABA inhibitors and 1 μM scopolamine, black traces), and after adding 6–8 μM carbachol (gray traces). **(C,D)** Blockade of nicotinic receptors by an agonist DMPP: **(C)** average EPSP amplitudes and **(D)** corresponding normalized EPSP amplitudes in control conditions with ACSF (black traces) and after application of 10 μM DMPP (gray traces). Data are expressed as mean ± SEM, *P ≤ 0.05, **P ≤ 0.01.

The addition of carbachol to the ACSF already containing the selective muscarinic receptor antagonist scopolamine (1 μM) resulted in only a very weak but still consistent and significant (*P* < 0.001, single group *t*-test) depolarization of 0.98 ± 0.12 mV (*n* = 9). Moreover, the presence of scopolamine prevented the EPSP amplitude reduction caused by carbachol. Instead, a weak increase in EPSPs amplitudes (Figure 3A, gray trace) was observed (see also Table 1A).

In the presence of scopolamine, application of carbachol did not change the facilitation of synaptic responses (Figure 3B and Table 1B). Similarly, data comparisons did not reveal any changes in the momentary facilitation (EPSP_*N*_/EPSP_*N–*__1_) during the train (Table 1C). Thus, blocking muscarinic receptors reversed the carbachol-induced pronounced reduction of EPSP amplitudes to a moderate enhancement as well as eliminated all changes in facilitation followed by the application of this general cholinergic agonist.

The weak increase in the amplitudes observed after application of carbachol, when muscarinic receptors had been blocked by scopolamine, could have been a result of the activation of nicotinic receptors. To verify this, another group of experiments using specific nicotinic agonist DMPP (10 μM) was performed. Activation of nicotinic receptors by DMPP led to a moderate depolarization in all investigated cells (*n* = 9) – on average by 3.78 ± 0.43 mV. In all cells treated with DMPP, amplitudes of the EPSPs became significantly larger (Figure 3C and Table 1A). The largest increase in the amplitude after application of DMPP was observed for the first EPSP (to, on average, 148% of control value). The 2nd, 3rd, 4th, and 5th EPSPs increased progressively less to 139, 129, 121, and 119% of control values.

Facilitation estimated from consecutive normalized amplitudes (EPSP_*N*_/EPSP_1_ – Figure 3D) decreased in the presence of DMPP (Table 1B). Thus, 10 μM DMPP increased EPSP amplitudes and at the same time reduced frequency-dependent facilitation of EPSPs in the train. The instantaneous facilitation (EPSP_*N*_/EPSP_*N–*__1_) decreased only between the first two postsynaptic responses.

Thus, selective activation of nicotinic receptors by 10 μM DMPP, moderately increasing EPSP amplitudes and decreasing their facilitation, seemed to have an opposite effect on corticothalamic synaptic transmission compared to carbachol. Since application of DMPP and application of carbachol after blocking muscarinic receptors by scopolamine had similar effects, we concluded that carbachol-induced reduction of EPSP amplitudes and enhancement of their facilitation are mediated by activation of muscarinic cholinergic receptors. The additional decrease of facilitation after specific nicotinic activation by DMPP may be due to the different strength by which 10 μM DMPP and 6–8 μM carbachol activate individual subtypes of nicotinic receptors. Application of 10 μM DMPP had in fact a larger depolarizing effect (3.78 ± 0.43 mV) than carbachol with blocked muscarinic receptors (0.98 ± 0.12 mV, *P* < 0.001).

### Carbachol-Induced Reduction of Corticothalamic EPSP Amplitudes and a Parallel Increase of Their Facilitation Are Associated With a Decreased Transmitter Release Probability

The coexistence of two effects caused by the application of carbachol, i.e., the depression of corticothalamic EPSP amplitudes and enhancement of their frequency-dependent facilitation suggests a presynaptic process underlying this general cholinergic modulatory action (Zucker and Regehr, 2002). To substantiate this finding, we used the “coefficient of variation (CV)” analysis of EPSP amplitudes (Clements, 1990). The CV method is based on the mathematical model describing the process of the neurotransmitter release formulated by del Castillo and Katz (1954). According to this model CV of postsynaptic response amplitudes depend only on two presynaptic factors: the probability of release of a neurotransmitter quantum – *q*, and the number of available units (quanta) – *n*, which has been correlated with the number of morphologically identified release sites or active zones (Korn et al., 1981, 1982; Korn and Faber, 1991). As both CV factors (*q* and *n*) characterize solely presynaptic mechanisms, any drug-related modulation of the postsynaptic site should not change the CV values of EPSP amplitudes (Clements, 1990). In contrast, a big difference in CV values before and after application of the tested drug strongly implicates a presynaptic site of the modulation.

**FIGURE 4:**
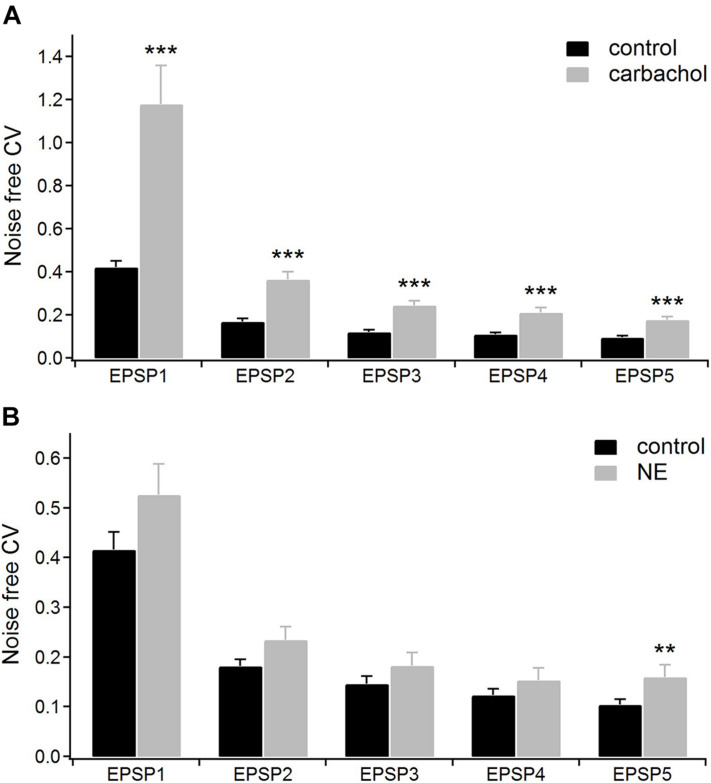
The coefficients of variations (CV) analysis. **(A)** Mean CV values (*n* = 16) for consecutive EPSPs of the train in the control condition (with ACSF containing GABA inhibitors) and after application of 6–8 μM carbachol. **(B)** Mean CV values (*n* = 15) for the consecutive EPSPs in the control condition (with ACSF containing GABA inhibitors plus ascorbic acid) and after application of 100 μM norepinephrine. Data are expressed as mean ± SEM, ***P ≤ 0.001, **P ≤ 0.01.

For the group of cells studied with carbachol (*n* = 16), we calculated the noise-free inter-trial CV values for consecutive EPSPs in the train (see section “Materials and Methods” for details) before and after application of the drug (Figure 4A). The average CV values became much higher after application of carbachol. The largest increase of CV value (2.8 times) was found for the 1st EPSP. The CV values calculated for the following EPSPs were about two times larger compared to the control condition (Figure 4A and Table 1A). Such a large increase of the CVs in the presence of carbachol strongly points to a presynaptic site of action of this drug (Clements, 1990; Nagumo et al., 2011).

To verify if the presynaptic mechanism of the cholinergic modulation relies on the decreased probability of transmitter release, PoM cells (*n* = 6) were studied during so-called “pseudominimal stimulation” of the corticothalamic fibers (see “Materials and Methods” for details). An increased failure rate of postsynaptic responses after application of carbachol would indicate reduced transmitter release probability caused by the drug and further support the presynaptic site of cholinergic modulation.

**FIGURE 5:**
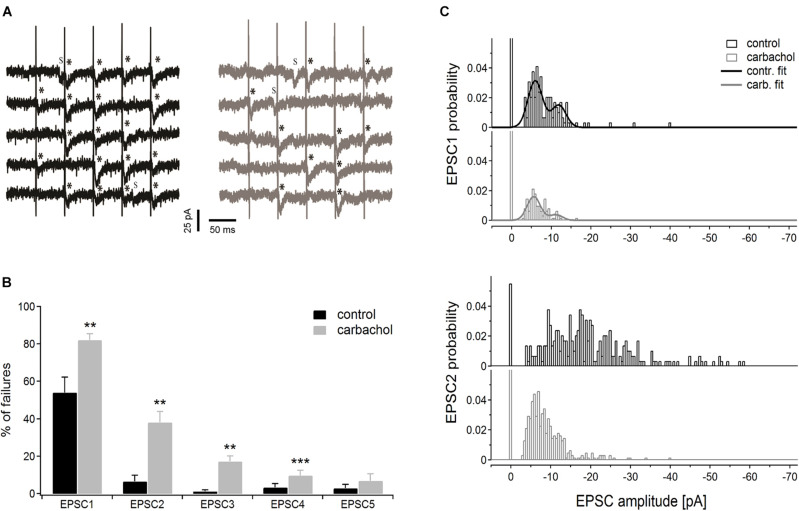
Pseudominimal stimulation experiment (*n* = 6 PoM cells). **(A)** Examples of the unitary excitatory postsynaptic currents (EPSCs) evoked in a single cell by trains of five pseudominimal stimulation of the corticothalamic fibers in the control condition (with ACSF containing GABA inhibitors, black traces on the left) and after application of 6–8 μM carbachol (gray traces on the right). Successively evoked EPSCs are marked with asterisks (*), letter “s” indicates the spontaneous EPSCs. **(B)** Percentage of the failures averaged for a group of cells in the control condition and after adding carbachol. **(C)** Histograms showing amplitude distribution for 1st (upper panel) and 2nd (lower panel) EPSCs in the train, in control conditions and with the addition of carbachol. Bars drawn at zero of the abscissa axes indicate probabilities of failures. Continuous black and gray curves on EPSC1 histograms show the appropriate double Gaussian fits. Data are expressed as mean ± SEM, **P ≤ 0.01, ***P ≤ 0.001.

Representative time courses of membrane currents recorded in a single cell during five consecutive stimulations in the control condition and after application of carbachol are shown in Figure 5A. Note, that the number of evoked EPSCs (marked by asterisks) was about two times higher (19 synaptic events in response to 25 stimulation pulses) in control conditions than during the recordings with the presence of cholinergic agent (10 events). Accordingly, the number of the failures was much smaller in the control state (6 vs. 15 in the presence of carbachol). Note that some spontaneous responses with amplitudes similar to evoked EPSCs were also recorded (Figure 5A, indicated by “S”). This observation indicated that only a small number of corticothalamic axons were stimulated. The average EPSC failure rate (i.e., number of failures divided by the number of stimulation trains and multiplied by 100%) for the 1st, 2nd, 3rd, and 4th EPSCs (Figure 5B) was substantially higher after application of carbachol (see also Table 1A). The failure rate of the 5th EPSC in the presence of carbachol was low and did not differ from the value in the control condition. In fact, the last (5th) stimulation pulse in the train typically produced the strongest and most reliable postsynaptic response.

We also investigated how carbachol affected the amplitude distribution of EPSCs (Figure 5C). The bimodal nature of the uppermost histograms for the 1st EPSC indicates that the first impulse in the train evoked EPSCs caused by the release of primarily two quanta. Comparison of amplitude histograms obtained for the 1st EPSC before and after application of carbachol suggests that the released quantal size was not affected by adding the drug. Although carbachol markedly reduced the total amount of EPSC responses (i.e., in control situation the probability of evoking an EPSC by the 1st impulse in the train was much larger) it did not change the positions of the two peaks of the histogram. To determine more precisely the quantal size of corticothalamic EPSCs, the histograms of amplitude probabilities obtained for the 1st impulse were fitted with a double Gaussian function (del Castillo and Katz, 1954; see section “Materials and Methods”). The fitting procedure returned the following quantal size values ± 95% confidence intervals: –5.9 ± 0.2 pA for the control situation and –5.6 ± 0.2 pA after addition of carbachol. As 95% confidence limits overlap, we can state that the obtained two quantal amplitudes are similar. This suggests that carbachol did not change the postsynaptic response size to the release of a single vesicle. This was most apparent for the first EPSC in the train but was also seen for the later EPSCs.

To conclude, results obtained during the pseudominimal stimulation directly indicated that cholinergic modulation of corticothalamic synapse formed by the axons from layer 6 on PoM neurons is presynaptic and relies on a decreased probability of transmitter release.

### Multiple Types of Adrenergic Receptors Mediate the Changes Caused by Application of Norepinephrine

**FIGURE 6:**
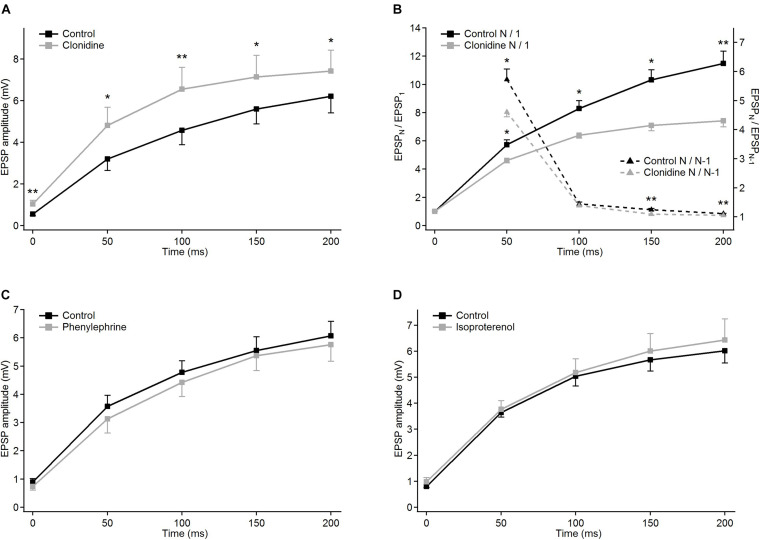
Modulatory effects of different noradrenergic agents on EPSP trains in PoM cells. Group average of EPSP amplitudes in the control condition (black traces) and after application of the drugs (gray traces): **(A)** 40 μM clonidine (*n* = 6); **(C)** 100 μM phenylephrine (*n* = 6) and **(D)** 100 μM isoproterenol (*n* = 7). **(B)** Clonidine effects on normalized EPSP amplitudes and momentary facilitation. Left *Y* axis: average normalized amplitudes (EPSP_*N*_/EPSP_1_) in the control condition (solid black trace) and in the presence of clonidine (solid gray line). Right *Y* axis: average ratios of the consecutive EPSP amplitudes (EPSP_*N*_/EPSP_*N–*__1_) in the control condition and after application of clonidine (black and gray dashed lines, respectively). For all NE drugs, control bath solution contained GABA inhibitors plus sodium ascorbate. Data are expressed as mean ± SEM, *P ≤ 0.05 and **P ≤ 0.01.

In the presence of noradrenergic agonist norepinephrine, a significant increase of the CV value was found only for the 5th EPSP (Figure 4B and Table 2A). These results do not distinctly support the hypothesis of the presynaptic mechanism of noradrenergic modulation. We suspected that this could be due to various effects exerted by different groups of norepinephrine receptors. Therefore, in the following experiments we investigated to what extent α-2, α-1, and β receptors were involved in noradrenergic modulation of the corticothalamic synapse from layer 6 to the PoM (Figure 6).

To check the involvement of α-2 receptors we studied the effect of clonidine on the amplitudes of evoked EPSPs. In contrast to norepinephrine, application of clonidine did not change the resting membrane potential of the investigated cells – the average change in the membrane potential was –0.58 ± 0.62 mV (not different from zero, *P* = 0.4, single group Student *t*-test). The plot of the average EPSP amplitudes obtained from a group of cells (*n* = 5) indicated that activation of α-2-adrenergic receptors increased amplitudes of the EPSPs (Figure 6A and Table 2A). On average, compared to the control values, clonidine increased the EPSP amplitudes by 1.89, 1.51, 1.43, 1.28, and 1.19 times respectively. Interestingly, the largest increase in the presence of clonidine was noted for the amplitude of the 1st EPSP. Hence, clonidine seemed to reduce the facilitation of the consecutive responses in the train. This is shown by the normalized amplitudes (EPSP_*N*_/EPSP_1_; Figure 6B and Table 2B) which had significantly larger values in control conditions than after application of clonidine. Ratios between amplitudes of neighboring EPSPs (see dashed lines on Figure 6B) were smaller in the presence of clonidine also for the 3rd (EPSP_4__/__3_) and 4th (EPSP_5__/__4_) pairs (Table 2C).

Summing up, the application of clonidine, similar to norepinephrine, decreased the frequency-dependent facilitation of the EPSPs evoked by the stimulation train. Thus, activation of α-2 adrenergic receptors replicated a part of the effects caused by norepinephrine. Moreover, the lack of membrane potential changes with application of clonidine suggested presynaptic action of α-2 receptors.

The difference between results obtained with the general adrenergic agonist norepinephrine (moderate decrease of EPSP amplitudes) and specific alpha-2 agonist clonidine (moderate increase of EPSP amplitudes) suggested that yet another class of adrenergic receptors, having a decreasing effect on EPSP amplitudes, should also be involved in the noradrenergic modulation of the corticothalamic synapses to the PoM.

Selective activation of α-1 receptors by phenylephrine led to the depolarization of the thalamic cells (*n* = 6) by 7.22 ± 1.11 mV on average. Phenylephrine did not cause any changes in the average EPSP amplitude values (Figure 6C and Table 2A). Consequently, the normalized amplitudes did not differ before and after α-1 adrenergic activation. Neither, phenylephrine changed the consecutive EPSP ratios. Thus, selective activation of α-1 adrenergic receptors did not affect EPSP amplitudes of PoM cells after activation of cortical layer 6 axons nor did it change the frequency-dependent facilitation, what suggested that α-1 receptors were not involved in noradrenergic modulation of the corticothalamic synapses. However, these receptors were partly responsible for the membrane potential shift caused by norepinephrine.

Finally, the role of the β receptors in the noradrenergic modulation was studied by application of a non-selective β-adrenoreceptor agonist isoproterenol. Activation of β adrenergic receptors depolarized the (*n* = 7) PoM cells on average by 7.34 ± 0.57 mV, but similarly to phenylephrine did not affect the amplitudes of the EPSPs in the train (Figure 6D). Similarly, the normalized amplitude values after β adrenergic activation did not change compared to the corresponding control values (Table 2B). Thus, selective activation of the β-adrenergic receptors did not affect the EPSP amplitudes in the train neither it changed their frequency-dependent facilitation. However, activation of this group of receptors resulted in the membrane potential shift which was about half of that seen after norepinephrine application.

## Discussion

This study provides the first data concerning cholinergic and noradrenergic modulation of the corticothalamic synaptic transmission from the cortical layer 6 to the cells in the higher-order posteromedial nucleus (PoM) of the somatosensory thalamus in mammals. We have characterized these modulations in rats PoM cells and showed that they substantially differ from each other.

Cholinergic modulation (induced by application of the general cholinergic agonist carbachol) led to a substantial decrease in PSPs amplitudes but at the same time enhanced frequency-dependent facilitation. This cholinergic modulation was caused by activation of muscarinic receptors, as it was reliably eliminated by muscarinic receptor blockage and did not appear in the presence of the agonists selective for nicotinic acetylcholine receptors. With cholinergic modulation, the amplitudes of consecutive EPSPs in the five pulse trains had a much higher trial-to-trial CV (SD/mean), suggesting a presynaptic change in the transmitter release probability rather than a postsynaptic change in the EPSP scaling. This was confirmed by increased failure rates to “pseudominimal” stimulation of the corticothalamic axons.

Noradrenergic modulation of the same synapse (mimicked by the application of general agonist norepinephrine) was different in all these respects. The amplitude of the first EPSP in the train was unchanged whereas amplitudes of the 2nd, 3rd, 4th, and 5th EPSPs decreased. In contrast to the cholinergic effect, the adrenergic activation decreased the frequency-dependent facilitation at the corticothalamic synapse. Norepinephrine did not change the coefficients of variation of consecutive EPSPs in the train in any consistent way. Thus, we could not find a support for either presynaptic or postsynaptic site of noradrenaline action.

### Receptors Regulating Cholinergic Modulation of Corticothalamic Transmission From Layer 6 to the PoM

In order to reveal what type of receptors are responsible for the cholinergic modulation of the corticothalamic transmission, we used drugs with selective pharmacological profiles. Application of carbachol after the pre-incubation with 1 μM scopolamine (a selective and powerful muscarinic antagonist) did not reduce the EPSPs and did not change the frequency-dependent facilitation. Instead, the responses were slightly increased in amplitude compared to the control condition. Therefore, we concluded that the modulatory action of carbachol on corticothalamic transmission in PoM was due to activation of muscarinic receptors and did not involve nicotinic receptors.

This conclusion was further supported by experiments with selective activation of nicotinic receptors by DMPP. There is a great diversity of nicotinic receptor subtypes depending on the α- and β-subunits composition, with DMPP affinities ranging from nanomolar to micromolar range (Parker et al., 2001; Romanelli et al., 2001). We decided to perform experiments with 10 μM concentration of DMPP as it should activate most of the nicotinic receptors and was comparable to the concentration of carbachol in the experiments with muscarinic receptors blocked by scopolamine. The effect of nicotinic receptors activation was, however, completely different than that of muscarinic receptors – the EPSP amplitudes were enhanced and frequency-dependent facilitation was reduced. The changes induced by DMPP were also small compared to those induced by carbachol. Our data were not sufficient to suggest the postsynaptic or presynaptic site of DMPP action. A possible presynaptic mechanism could rely on an increase of the probability of transmitter release. However, we do not exclude that any postsynaptic mechanisms could also be involved in the observed modulation (Blitz et al., 2004; Sun and Beierlein, 2011), although it should not depend on changes of the membrane resistance, as it did not change after incubation with the drug.

Thus, our results indicate that carbachol-induced depression of the EPSPs and simultaneous enhancement of the frequency-dependent facilitation of the corticothalamic input from the layer 6 to the PoM are caused by activation of muscarinic receptors. These effects are accompanied by smaller nicotinic modulation acting in the opposite direction. This smaller modulation was not visible after general cholinergic activation, presumably being hidden by an overwhelming muscarinic effect. More extensive studies are needed to reveal the role and mechanism of this weaker nicotinic effect.

Our study provides the first data concerning the cholinergic modulation of the corticothalamic synaptic input to the mammalian higher-order sensory thalamic nuclei. Similar experiments have been conducted in the first order ventrobasal (VB) nucleus of mouse. In general, these results were similar: postsynaptic responses were decreased and simultaneously, the frequency-dependent facilitation was enhanced (Castro-Alamancos and Calcagnotto, 2001; Nagumo et al., 2011). In addition, these studies showed that types of receptors involved in such modulatory effects depend on the age of the animals. In young adult (>7-week old) mice these effects were mediated by muscarinic receptors (Castro-Alamancos and Calcagnotto, 2001), while in neonatal (14–19 days old) mice they were mediated by nicotinic receptors, particularly by those containing the α-5 subunit (Nagumo et al., 2011). Nagumo et al. (2011) proposed that this age-dependent difference may be caused by developmental changes in the expression of acetylcholine receptors during the postnatal development. In particular, nicotinic receptor expression usually decreases, and muscarinic receptor expression increases during the postnatal development in mice (Fiedler et al., 1987). The rats we used were in the middle of this age range (3–4 weeks old, i.e., weaning age) but the postnatal development of the cholinergic receptors may slightly differ between rats and mice or between first order and second order thalamic nuclei. These observations should be taken into account when accepting the major muscarinic nature of the cholinergic modulation found in our study.

Despite similar effects (depression of the postsynaptic responses and enhancement of facilitation) induced by cholinergic agents in the primary and secondary relay nuclei in both young and adult rodents, the subtypes of the receptors (muscarinic or nicotinic) involved in these processes might differ. We did not examine the involvement of particular subtypes of muscarinic receptors (M1–M5), mainly because of the lack of highly specific agonists and antagonists. We suppose, however, that M2 receptors could be involved in the cholinergic modulation. First of all, the affinity of carbachol to M2 receptors is higher than to other muscarinic receptor types (Peralta et al., 1987; Jakubik et al., 1997; Cheng et al., 2002) and these receptors are located on the presynaptic terminals (Guo et al., 2012). Moreover, higher-order nuclei in adult rats contain more muscarinic M2 receptors compared to the first-order nuclei (Barthó et al., 2002). However, one cannot exclude either that more than one subtype of muscarinic receptors may be involved in the processes of cholinergic modulation in PoM.

### Mechanism of Cholinergic Modulation of Corticothalamic Transmission From Layer 6 to the PoM

We also aimed to study whether cholinergic modulation is supported by pre- or postsynaptic mechanism. Simultaneous decrease of the EPSP amplitudes and enhancement of the frequency-dependent facilitation induced by carbachol are consistent with a presynaptic mechanism related to the decrease of the neurotransmitter release probability (Zucker, 1989; Zucker and Regehr, 2002). This hypothesis posits that a low initial release probability initiates stronger facilitation of the postsynaptic responses (Manabe et al., 1993). To solve this issue, we used an analysis based on the CV which has been used previously to study the site of the action of a modulatory drug (Clements, 1990; Faber and Korn, 1991; Hannay et al., 1993; Sjöström et al., 2003). As the inter-trial, noise-free CV values for all EPSPs in the train were much larger after cholinergic activation we assumed that a presynaptic mechanism was responsible for the carbachol-induced cholinergic modulation.

Direct experimental evidence for such presynaptic modulatory action of carbachol was obtained by measuring the unitary EPSCs using a pseudominimal stimulation of the corticothalamic tract. Activation of only one corticothalamic axon (Hanse and Gustafsson, 2001; Granseth and Lindström, 2003) is very difficult since the synaptic transmitter release probability is exceedingly small (<10%; Granseth and Lindström, 2003). However, for establishing if a drug effect is pre- or postsynaptic, an EPSC failure rate analysis can be performed with less strict experimental conditions. EPSC failures are seen in the postsynaptic cell when the action potential does not release neurotransmitter and is related to the transmitter release probability (p) and the number of release sites (n) as (1–p^*n*^). An increase in the number of EPSC failures would consequently represent a reduction in the transmitter release and *vice versa*. Thus, the most sensitive way to probe for a change in presynaptic transmitter release was to adjust the stimulation pulse intensity to have 50% of EPSCs failures. We called this “pseudominimal” stimulation since more than one axon was recruited by the stimulation pulses. Our results showed that for each of four first impulses in the train carbachol caused a substantial increase in the number of failures which clearly indicated a decrease of transmitter release probability. The facilitation mechanism of the studied synapse substantially increased the probability of transmitter release during the train and carbachol-induced reduction of the failures was not significant for the 5th EPSC. This did not necessarily mean that carbachol did not reduce the release probability for the last stimulus (for which the averaged EPSP amplitude still remained lower under carbachol). The EPSC amplitude histograms showed, in addition, that carbachol did not change the unitary size of the postsynaptic responses caused by a single synaptic vesicle and provided a further support for the presynaptic site of modulatory action.

Taken together our data demonstrate that carbachol exerts presynaptic modulatory action on corticothalamic synaptic transmission from layer 6 of area S1 to PoM neurons by decreasing the probability of transmitter release. There is no evidence that cholinergic synapses are located directly on the corticothalamic connections, but acetylcholine could activate presynaptic muscarinic cholinergic receptors located at the synapses by means of volume transmission. It was previously established that a decrease of the initial release probability led to an enhanced facilitation (Manabe et al., 1993). Such a mechanism might explain the increase of carbachol-induced facilitation of the subsequent corticothalamic responses if the spikes arrive sufficiently close to each other.

Suppression of the glutamate release by activation of muscarinic receptors was previously suggested for many other synaptic connections (Williams and Johnston, 1990; Sim and Griffith, 1996; de Sevilla et al., 2002; Zhang and Warren, 2002; Guo et al., 2012). It is also known that presynaptic action of cholinergic agents decreases excitatory transmission in various other structures such as hippocampus (Scanziani et al., 1995), ventral striatum (Pennartz and Lopes da Silva, 1994) and, interestingly, inhibitory transmission in the thalamus (Masri et al., 2006). Other experiments suggest (by indirect effect of the enhanced frequency-dependent facilitation) that carbachol decreases the transmitter release probability at the corticothalamic synapses also in the first order VB complex (Castro-Alamancos and Calcagnotto, 2001; Nagumo et al., 2011).

Although our data strongly supports the involvement of presynaptic muscarinic receptors in cholinergic modulation of corticothalamic transmission to the PoM, we do not know which elements from the cascade of the events leading to the release of the neurotransmitter are actually affected by this modulatory process. In general, two types of events at the corticothalamic terminal can be regulated: calcium entry through voltage-gated calcium channels and the factors responsible for the preparation of the release-ready vesicles and their final exocytosis. For example, in case of presynaptic muscarinic inhibition of the excitatory synaptic transmission in CA3 area of hippocampus (Scanziani et al., 1995) the results suggested direct interference in the neurotransmitter release process at some point subsequent to calcium influx. It remains an intriguing question whether this might also be true also in the rat’s PoM.

It is important to mention that we cannot exclude other postsynaptic mechanisms like receptor saturation or desensitization to be involved in muscarinic modulation in PoM. It has been shown that postsynaptic mechanisms can affect the frequency-dependent facilitation of postsynaptic responses (Blitz et al., 2004; Sun and Beierlein, 2011) and one of these processes – receptor saturation was acknowledged in corticothalamic synapses in the first order VB complex of mice (Sun and Beierlein, 2011). It is likely that such a postsynaptic mechanism can additionally shape the muscarinic modulation. Following synaptic depression, the smaller amounts of neurotransmitter released into the synaptic cleft will have less chance to saturate the postsynaptic receptors. As a consequence, less saturation would additionally raise the facilitation enhancement at the presynaptic site. Further experiments are needed to investigate other postsynaptic mechanisms that may also be involved in the cholinergic modulation of the corticothalamic synapses in PoM.

### Mechanism Underlying the Noradrenergic Modulation of the Corticothalamic Transmission From Layer 6 to the PoM

Activation of noradrenergic receptors by noradrenaline led to the depression of the later EPSP amplitudes with an unchanged magnitude of the 1st EPSP and reduced frequency-dependent facilitation during the EPSP train. Closer inspection of the ratios between the amplitudes of the consecutive postsynaptic responses showed that the decreased facilitation resulted solely from the difference between the first two EPSPs (Figure 2F). The observed effects of noradrenergic modulation of synaptic transmission from layer 6 to the PoM were surprising for us. Assuming that a presynaptic mechanism is at work, i.e., by changing the initial transmitter release probability, a decreased facilitation should have led to a larger amplitude of the 1st EPSP (Zucker, 1989; Zucker and Regehr, 2002). However, the 1st EPSP in the presence of norepinephrine was not changed. One should, therefore, consider a possible mixture of the effects caused by different subtypes of adrenergic receptors or that both pre- and postsynaptic sites may be involved in the noradrenergic modulation or a direct effect on the facilitation mechanism *per se*.

A similar conclusion can be drawn from the analysis of coefficients of variation. In contrast to the cholinergic modulation, where CVs for all the EPSPs were much larger after application of carbachol, norepinephrine did not cause a consistent change in CV values. Such a result does not support to any change in the transmitter release probability.

To better understand the process of noradrenergic modulation in PoM, we selectively activated the α-2 adrenergic receptors using specific agonist clonidine. The reason for performing this experiment was that α-2 receptors were shown to be involved in noradrenergic modulation of the corticothalamic transmission to VB in mice (Castro-Alamancos and Calcagnotto, 2001). In our experiments, clonidine did not depolarize PoM cells, which was in accordance with their putative presynaptic localization. Moreover, activation of α-2 receptors increased the amplitudes of all EPSPs, including first, and lowered the frequency-dependent facilitation during the train. This fits the classical picture observed after an increase in transmitter release probability.

It should be noted that clonidine appeared to increase the EPSP amplitudes, which is opposite to the effect of the general agonist norepinephrine. Most probably, another group of adrenergic receptors (α-1 or β) substantially depressed the corticothalamic postsynaptic responses in PoM and the reduction with noradrenergic effect is the net effect of all these receptors being activated together. However, activation of α-1 adrenoceptors by application of phenylephrine had no effect on the EPSP amplitudes or the frequency-dependent facilitation at the corticothalamic synapse. This data indicates that these receptors are not involved in the modulation of the corticothalamic synaptic transmission to the PoM by norepinephrine despite the fact that they had a consistent depolarizing postsynaptic effect on all studied PoM cells.

It was previously found that β adrenergic receptors can affect short term synaptic properties (Pu et al., 2009). However, we did not find experimental proof that β-adrenoceptors are responsible for the noradrenergic modulation of corticothalamic synaptic transmission in PoM. Although isoproterenol, a general β-receptor agonist (Baker et al., 1991) similar to phenylephrine, consistently depolarized the cells it had no effect on the response amplitudes neither it affected their frequency-dependent facilitation. Differences between the affinities of isoproterenol and norepinephrine to different subclasses of adrenergic β receptors might, to some extent, explain this discrepancy. Namely, isoproterenol has a greater affinity to both β-1 and β-2 adrenergic receptors as compared to norepinephrine (Sillence et al., 2005). This compound was also found to be equally potent on β-1 and β-2 adrenergic receptors, while norepinephrine is 10-fold more selective for β-1 than for β-2 receptors (Michel, 1991; Hoffmann et al., 2004). Thus, further experiments with the use of more selective β-1 and β-2 receptor agonists might finally reveal the receptors underlying the noradrenergic modulation of the corticothalamic synapses to the PoM. Finally, the modulatory effect of norepinephrine might be not a simple summation of the separate actions produced by more specific agonists. When activated simultaneously, different adrenergic receptor subtypes could interact to shape the response in different ways.

Noradrenergic modulation of corticothalamic synaptic transmission was investigated before by Castro-Alamancos and Calcagnotto (2001) in the first order ventrobasal (VB) nucleus of mice. These authors revealed that both noradrenergic and cholinergic activation decreased the postsynaptic responses with a simultaneous increase of the frequency-dependent facilitation at the synapse. The noradrenergic modulation was shown to be mediated by α2-adrenergic receptors and the authors proposed that the mechanism of this synaptic regulation was presynaptic. Our results also show that α2-adrenergic receptors modulate layer 6 input to higher-order PoM nucleus of the rat, but in the opposite direction – as compared to the VB of mice - by enhancing synaptic responses and decreasing their frequency-dependent facilitation. The difference may be related to different species used, different nuclei which were investigated or different ages of experimental animals (adult, older than 7 weeks mice versus 3–4 weeks old rats). Age-related differences in the noradrenergic modulation would be possible because of temporal differences in the postnatal development of adrenergic receptors (Happe et al., 2004) and could resemble age-related differences in the cholinergic modulation in VPM of mice (Castro-Alamancos and Calcagnotto, 2001 vs. Nagumo et al., 2011).

Interestingly, some developmental changes in noradrenergic modulation occur also within the cortex in the case of the layer 5 corticothalamic neurons. Such cells in juvenile rats (3–4 weeks old, as in our study) have almost exclusively regular spiking firing pattern, while in adults predominantly show a bursting activity (Llano and Sherman, 2009). In parallel, norepinephrine enhances synaptically driven responses in regularly spiking layer 5 cells but depresses them in bursting neurons (Waterhouse et al., 2000). In consequence, synaptic responses of layer 5 corticothalamic cells can be enhanced in juvenile but depressed in adult rats. The maturation of noradrenergic modulation of layer 6 synaptic input to the PoM could go hand in hand with age-related noradrenergic effect within the layer 5.

Taken together, our data provide an evidence that noradrenergic modulation of layer 6 corticothalamic transmission in PoM acts (at least partly) via the α-2 receptors. Additional experiments are needed to reveal all the receptors and mechanisms involved in this process.

### Functional Role of Cholinergic and Noradrenergic Modulation of Corticothalamic Transmission From Layer 6 to the PoM

Cholinergic and noradrenergic connections in the brain form rich, complex, and mutually linked neuromodulatory system playing an important role in the transition from sleep to arousal, setting different levels of vigilance, attentive behavior or executive function. The classical experiment by Livingstone and Hubel (1981) showed that the activity of cells in the cortical layer 6 is profoundly depressed during sleep and activated during arousal evoked by brainstem stimulation. The regulation of arousal is provided by cholinergic afferents from the brainstem pedunculopontine and laterodorsal tegmental nuclei to the thalamo-cortical system (Steriade et al., 1993; Pita-Almenar et al., 2014; Trofimova and Robbins, 2016) whereas the afferents from the basal forebrain to cortical and some thalamic sites (Varela, 2014) participate in the regulation of attentive processes induced by a novel, salient or “emotionally charged” stimuli (Klinkenberg et al., 2011; Unal et al., 2012). In parallel, noradrenergic afferents from the locus coeruleus have strong reciprocal connections with the prefrontal cortex, are activated by important, salient stimuli, and initiate attentive processing (for reviews see: Sarter and Bruno, 2000; Samuels and Szabadi, 2008; Sara, 2009).

We have previously proposed that the functional role of the frequency-dependent facilitation at the corticothalamic synapse might be to provide a dynamic gain control of the transmission of the sensory information through the thalamus (Lindström and Wróbel, 1990; Granseth et al., 2002; Granseth, 2004). Later results carried out in our laboratory (Bekisz and Wróbel, 1993; Wróbel et al., 2007) showed that this gain enhancement operates in the beta frequency band (12–30 Hz) and may be utilized as an attentional mechanism. It was hypothesized that short-lasting (200–300 ms) beta oscillatory bursts in the corticothalamic pathway can depolarize the thalamic neurons by means of frequency-dependent facilitation and thus change the gain for the information stream from the periphery to the cortex (Wróbel, 2000, 2014). Activation of the cholinergic (and/or noradrenergic) system could provide further control of this gain mechanism (Wróbel and Kublik, 2001).

It has been previously shown that activation of both cholinergic and noradrenergic systems increases the frequency-dependent facilitation in the first order, VPM nucleus (Castro-Alamancos and Calcagnotto, 2001). However, *in vivo* cholinergic activation increases the spontaneous firing and enlarges the VPM receptive fields, whereas noradrenergic activation decreases spontaneous activity and focuses the receptive fields (Hirata et al., 2006). It was proposed that the two modulatory systems play different roles in information processing at the first order somatosensory thalamus, with noradrenergic modulation being more specific/focused than cholinergic (Hirata et al., 2006).

Our data extends the notion, that in the higher-order PoM nucleus these two systems act differently – the cholinergic system enhances the frequency-dependent facilitation, while noradrenergic system reduces it. Interaction between the two systems is not yet understood. One has to take into consideration the complicated modulatory network acting on the secondary order nuclei. For example, it has been shown that cholinergic activation of *zona incerta* (Masri et al., 2006) increases the gain of information flow through the PoM. It is possible that reduction of the corticothalamic facilitation by noradrenaline counteracts this gain increase to keep the necessary balance of the activation in PoM. Whether this hypothesis survives the experimental investigation remains to be checked. Our experiment allows, however, to conclude that both cholinergic and noradrenergic modulation act as a variable dynamic control for the corticothalamic mechanism of the frequency-dependent facilitation in PoM.

## Data Availability Statement

The original contributions presented in the study are included in the article, further inquiries can be directed to the corresponding author/s.

## Ethics Statement

The animal study was reviewed and approved by 1st Local Ethic Commission in Warsaw and Committee for Ethics in Animal Research of Linköping in accordance with Polish, Swedish and EU legislations.

## Author Contributions

AW, MB, and BG conceived and designed the experiments. SN and MB performed the experiments in the Laboratory of Visual Neurobiology at the Nencki Institute of Experimental Biology of PAS. SN and BG carried on the experiments in the Department of Clinical and Experimental Medicine of Linköping University. SN, MB, and BG analyzed the data. SN, MB, EK, AW, and BG participated in the discussion of results and wrote the manuscript. All the authors approved the final version of the manuscript.

## Conflict of Interest

The authors declare that the research was conducted in the absence of any commercial or financial relationships that could be construed as a potential conflict of interest.
